# Bacterial community structure and co-occurrence networks in the rhizosphere and root endosphere of the grafted apple

**DOI:** 10.1186/s12866-024-03210-x

**Published:** 2024-02-10

**Authors:** Hui Cao, Longxiao Xu, Jianfei Song, Mi Xun, Weiwei Zhang, Hongqiang Yang

**Affiliations:** 1 College of Life Sciences, Zaozhuang University, Zaozhuang, 277000 Shandong Province China; 2 College of Horticulture Science and Engineering, Shandong Agricultural University, State Key Laboratory of Crop Biology, Tai’an, 271018 Shandong Province China

**Keywords:** Rhizosphere, Root endosphere, Bacterial community diversity, Co-occurrence network

## Abstract

**Background:**

Compared with aerial plant tissues (such as leaf, stem, and flower), root-associated microbiomes play an indisputable role in promoting plant health and productivity. We thus explored the similarities and differences between rhizosphere and root endosphere bacterial community in the grafted apple system.

**Results:**

Using pot experiments, three microhabitats (bulk soil, rhizosphere and root endosphere) samples were obtained from two-year-old apple trees grafted on the four different rootstocks. We then investigated the bacterial community composition, diversity, and co-occurrence network in three microhabitats using the Illumina sequencing methods. Only 63 amplicon sequence variants (ASVs) out of a total of 24,485 were shared in the rhizosphere and root endosphere of apple grafted on the four different rootstocks (M9T337, *Malus hupehensis* Rehd., *Malus robusta* Rehd., and *Malus baccata* Borkh.). The core microbiome contained 8 phyla and 25 families. From the bulk soil to the rhizosphere to the root endosphere, the members of the phylum and class levels demonstrated a significant enrichment and depletion pattern. Co-occurrence network analysis showed the network complexity of the rhizosphere was higher than the root endosphere. Most of the keystone nodes in both networks were classified as Proteobacteria, Actinobacteriota and Bacteroidetes and were low abundance species.

**Conclusion:**

The hierarchical filtration pattern existed not only in the assembly of root endosphere bacteria, but also in the core microbiome. Moreover, most of the core ASVs were high-abundance species, while the keystone ASVs of the network were low-abundance species.

**Supplementary Information:**

The online version contains supplementary material available at 10.1186/s12866-024-03210-x.

## Background

Apple is one of the fruits with the most widely cultivated acreage in the world [1], and its cultivated soil types and rootstock types are varied, which leads to the diversity of apple root morphology and root-zone soil microbiomes. These differences are crucial significance for apple to absorb and utilize nutrients from the soil and promote the improvement of fruit yield and quality.

The root, which plays a crucial role in the interaction between soil microbiomes and plants, is the basis of the plant. Root tissues provide colonization site and secrete essential organic compounds to maintains the plant-specific microbiota of root-zone soils [2, 3], which includes not only soil attached microorganisms adhering to the roots and inhabit the root surface, but also the colonized microorganisms in the root interior environment [4]. Depending on the chemical signals and nutrients released by the root, microorganisms are enriched and grown in the rhizosphere soil, and then they pass through the regulation and selection by plant own metabolism to stably colonize the root tissues [5–7].

Root-associated microbiomes mainly include viruses, bacteria, archaea, protozoa, and fungi [8]. Among them, the diversity and abundance of bacteria are enormous, and they play a more important role in promoting plant health and improving crop productivity [9]. For instance, the rhizosphere bacterial community can promote the decomposition of mineral nutrients, defend against soil-borne diseases and improve plant resilience to adverse growth conditions [10–12]. It is reported that the inoculated plant-growth-promoting rhizobacteria strains contributed to the increase in young apple tree growth and fruit yield [13]. Often the beneficial effects of root endophytes, without causing any evident damage to the host plants, are greater than many rhizosphere bacteria [5, 14]. Plant-growth-promoting bacterial endophytes facilitate plant growth by producing phytohormones, antimicrobial metabolites, and increasing supply of nutrients [15, 16].

In apple orchards, the roots of the rootstocks uptake nutrients from soil for the plant and are the primary site of rhizosphere microorganisms [2]. Chai et al. (2020) used Illumina MiSeq sequencing to determine the bacterial community of the rhizosphere from different rootstocks, they found apple rootstocks with different phosphorus efficiency showed alteration of the microbes in rhizosphere [17]. They also found the rhizosphere bacterial community structure significantly differed among the apple rootstocks of different nitrogen tolerance, for example, the bacterial phyla Proteobacteria and Actinobacteria were the dominant groups in the rhizosphere and presented higher abundance in the low nitrogen-tolerant rhizosphere [18]. Liu et al. (2022) also demonstrated a clear impact of root genotype on microbial composition and diversity [19]. Previous studies have indicated that the apple rootstock also has an important effect on the endophytic microbiota of different rootstock/scion combinations, interestingly, “M.M.111” rootstock with weak growth control properties had more beneficial and growth promoting fungal and bacterial taxa than “M.9” rootstock with strong growth control properties [20]. As reviewed by previous studies, rootstock genotypes can influence the taxonomy, structure,composition and network properties of the rhizosphere bacterial community in grapes [21, 22]. However, studies unveiling the bacterial community structure and network in the rhizosphere and root endosphere of grafted apple are lacking. In this study, we use a grafted apple system with four different rootstocks to study root-associated bacterial communities by 16S rRNA gene high-throughput sequencing. We compared the changes of the bacterial community diversities and co-occurrence network in the bulk soil, rhizosphere, and root endosphere. Our results will lay the groundwork for regulating the rhizosphere and root endosphere microbiomes to promote apple healthy growth.

## Materials and methods

### Experimental materials and design

The 2-year-old apple scion variety (*Malus domestica* Borkh.cv.Red Fuji), grafted on four rootstocks (M9T337, *Malus hupehensis* Rehd., *Malus robusta* Rehd., and *Malus baccata* Borkh.) were used in the study. The native soil from an arable site in Taian city (36°10′N, 117°07′E), Shandong Province, China, was collected at 0–20-cm depth. The soil is loam (21% clay, 29% powder and 50% sand) with a pH of 6.7, bulk density of 1.37 g·cm^−3^, available nitrogen of 80.50 mg·kg^−1^, available phosphorus of 66.46 mg·kg^−1^, available potassium of 129.84 mg·kg^−1^, organic matter of 10.05 g·kg^−1^ and it is classified as a Cinnamon soil. The experiment used a potted method, four different grafted seedlings were planted in pots with three replicates established in a completely randomized block design.

### Sample collection of the rhizosphere, root endosphere, and bulk soil

We separated the rhizosphere soil from the root endosphere according to the methods described previously [23]. Briefly, roots were manually removed from the pot using sterile gloves and gently shaken to remove loose soil. Root segments with adhering soil of 8 cm starting 2 cm below the root base were dissected with a sterile scalpel and placed into sterile tubes containing PBS-S buffer (130 mM NaCl, 7 mM Na_2_HPO_4_, 3 mM NaH_2_PO_4_, pH 7.0, 0.02% Silwet L-77). The root segments were washed twice with shaking at 180 rpm for 20 min each time, and the two washing buffers were combined. The pellet resulting from the centrifugation of the washing buffer for 20 min at 4000 g was defined as the rhizosphere samples and frozen for storage at − 80 °C.

The treated root segments were washed with water and moved to a new sterile tube. Next, the root segments were sterilized with 70% alcohol and a sodium hypochlorite solution containing 2.5% active Cl^−^, as described in Sun et al. [24]. Finally, the root segments were rinsed in sterile, distilled water several times. The sterile root segments were defined as the root endosphere samples and frozen for storage at–80 °C.

The bulk soil samples were collected from unplanted apple tree pots and the soil depth from 2 to 10 cm from the surface corresponding to 8 cm root length, then stored at − 80 °C until further processing.

### DNA extraction

Microbial community genomic DNA from the bulk soil, rhizosphere, and root endosphere samples were extracted using the E.Z.N.A.® Soil DNA Kit (Omega, USA) according to the manufacturer’s instructions. Then, total DNA was detected on 0.8% agarose gel electrophoresis and a Nanodrop 2000 UV-vis Spectrophotometer (Thermo Scientific, Wilmington, USA) was used to determined DNA concentration and quality.

### PCR amplification and sequencing

The V3-V4 hypervariable region of the bacterial 16S rRNA gene was amplified with primer pairs 338F (forward primer 5′-ACTCCTACGGGAGGCAGCA-3′) and 806R (reverse primer 5′-GGACTACHVGGGTWTCTAAT-3′). The PCR mixtures contain 5 × reaction buffer 5 μL, 5 × GC buffer 5 μL, dNTP (2.5 mM) 2 μL, Q5 DNA Polymerase 0.25 μL from Q5® High-Fidelity DNA Polymerase (New England Biolabs [NEB], MA, USA), forward primer (10 μM) 1 μL, reverse primer (10 μM) 1 μL, template DNA 2 μl, and finally ddH_2_O up to 25 μL. The PCR conditions 98 °C for 2 min, followed by 30 cycles of 98 °C for 15 s, 55 °C for 30 s, 72 °C for 30 s, and a final extension at 72 °C for 5 min. PCR reactions were performed in triplicate. The amplified PCR products were separated on 0.8% agarose gels, purified using an AxyPrep DNA Gel Extraction Kit (AXYGEN, USA) and quantified using a Quant-iT PicoGreen dsDNA Assay Kit and Microplate reader (BioTek, FLx800). Briefly, After obtaining the pure purified amplicons, we used the TruSeq® DNA PCR-Free Sample Preparation Kit (Illumina, USA) for library construction according to the manufacturer’s protocol. Nuclease free water (QIAGEN, Valencia, CA, USA) replaced template DNA in negative controls. The library quality was preliminary determined by Qubit® 2.0 Fluorometer (Thermo Scientific, USA), and Q-PCR according to Wang et al. (2022) was used for accurate and quantitative library detection [25]. After the library was qualified, the bacterial communities of all samples including negative controls were sequenced using the Illumina Miseq System by Personal Biotechnology Co., Ltd. (Shanghai, China). All sequence data have been deposited into the NCBI Sequence Read Archive database under accession number SRP280070.

### Processing and analysis of sequencing data

The raw data were performed using QIIME 2 version 2023.2. Briefly, raw sequence data were demultiplexed using the demux plugin, and primers were cut with cutadapt plugin. Sequences were then quality filtered, denoised, chimera removed and merged amplicon sequence variants (ASVs) using the DADA 2 plugin [26]. Finally, singletons ASVs were removed, and the sequencing depth of per sample was rarefied to counts up to 92,636 reads (the lowest sequencing depth of all samples). The taxonomy annotation of each ASVs representative sequence was analyzed using the Greengenes2 database (http://greengenes.secondgenome.com/ )[27].

### Statistical analysis

The α-diversity indexes (Chao1, Observed ASVs, Shannon, and Simpson) and rarefaction curves were evaluated by QIIME 2. The relationship between bacterial community structures of different samples was visualized using a principal coordinate analysis (PCoA) and clustering analysis based on Bray-Curtis distances. The Venn-diagram analysis was performed to calculate the shared ASVs among the rhizosphere and root endosphere (http://bioinformatics.psb.ugent.be/webtools/Venn/). The Sankey plots were performed in R version 3.6.1 using the network D3 package. According to the described previously [28], the significant differential abundance of bacteria at phylum and genus levels were performed using the STAMP software by Welch’s test. Statistically significant difference differences in α-diversity indexes between rhizophere, endosphere and microhabitat were assessed using Kruskal-Wallis test. The Stats package (R version 3.6.1) was used to perform the Mann-Whitney-Wilcoxon test.

To explore the interaction between the root-associated bacteria of grafted apple, the ASVs that had average relative abundance > 0.05% and presented in 50% of the samples were selected for Co-occurrence network analyses. Based on Random Matrix Theory (RMT) approach, the Co-occurrence networks of the rhizosphere and root endosphere bacteria were constructed using the Molecular Ecological Network Analyses Pipeline (MENA) (http://ieg4.rccc.ou.edu/mena) at the ASV level [29]. We screened for significant congruent pairs of rhizosphere and root endosphere bacteria based on the statistical significance (*P* < 0.05) and strength (ρ > 0.9) of the correlation. The visualization of the Co-occurrence networks was performed by Cytoscape version 3.7.2. According to the described previously [29, 30], the topological roles of each node can be defined by its within-module connectivity (Zi) and among-module connectivity (Pi). The keystone nodes (species) contain three types: network hubs (Zi  > 2.5 and Pi > 0.62) and module hubs (Zi > 2.5 and Pi ≤0.62).

## Results

### MiSeq sequencing data and quality

**Fig. 1:**
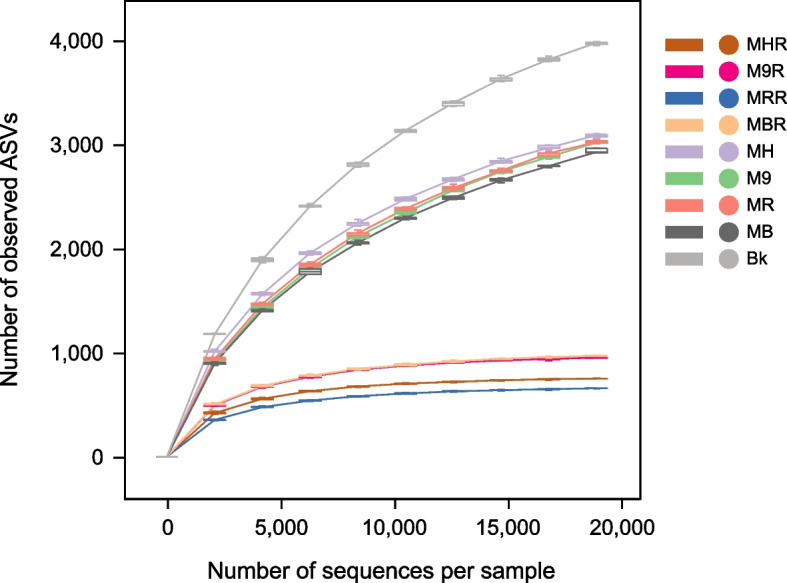
Rarefaction curves of the number of ASVs for each sample. MH, M9, MB and MR represent the rhizosphere from *Malus hupehensis* Rehd., M9T337, *Malus baccata* Borkh. and *Malus robusta* Rehd., respectively. MHR, M9R, MBR and MRR represent the root endosphere from *Malus hupehensis* Rehd., M9T337, *Malus baccata* Borkh. and *Malus robusta* Rehd., respectively. Bk represents bulk soil

Illumina Miseq sequencing of 16S rRNA gene results showed that a total of 3,376,644 effective sequences and 24,485 ASVs were identified in the bulk soil, rhizosphere and root endosphere of apple grafted on four different rootstocks. The number of ASVs was 8588, 15,179 and 5806 in the bulk soil, rhizosphere and root endosphere, respectively. The rarefaction curves approached the saturation plateau and good’s coverage was between 91.8–99.9% in all samples, suggesting that the sequencing depth was sufficient to reflect the bacterial community diversity in all samples (Fig. 1, Supplementary Table S1).

### Unique and common ASVs in the root systems of apple grafted on four different rootstocks

**Fig. 2:**
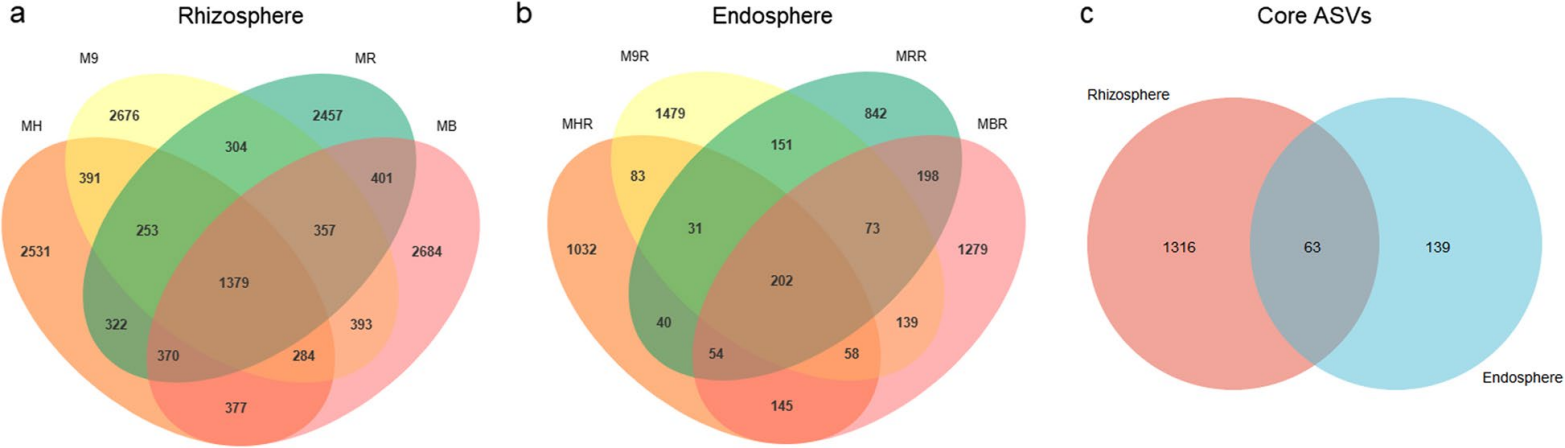
Venn diagram based on the number of ASVs associated with the rhizosphere and root endosphere of grafted apple. **a** Common and unique ASVs in the rhizosphere of four different rootstocks. **b** Common and unique ASVs in the root endosphere of four different rootstocks. **c** Core ASVs between rhizosphere and root endosphere of four different rootstocks. MH, M9, MB and MR represent the rhizosphere from *Malus hupehensis* Rehd., M9T337, *Malus baccata* Borkh. and *Malus robusta* Rehd., respectively. MHR, M9R, MBR and MRR represent the root endosphere from *Malus hupehensis* Rehd., M9T337, *Malus baccata* Borkh. and *Malus robusta* Rehd., respectively

Based on a Venn diagram analysis, the average number of unique ASVs associated with grafted apple rhizosphere (*n* = 2587) (Fig. 2a) was higher than the root endosphere (*n* = 1158) (Fig. 2b) regardless of the rootstock types. The number of unique rhizosphere ASVs in four different rootstocks showed a trend of MB > M9 > MH > MR (*n* = 2684, 2676, 2531 and 2457, respectively), and the root endosphere showed a trend of M9R > MBR > MHR > MRR (*n* = 1479, 1279, 1032 and 842, respectively) (Fig. 2a-b).

**Fig. 3:**
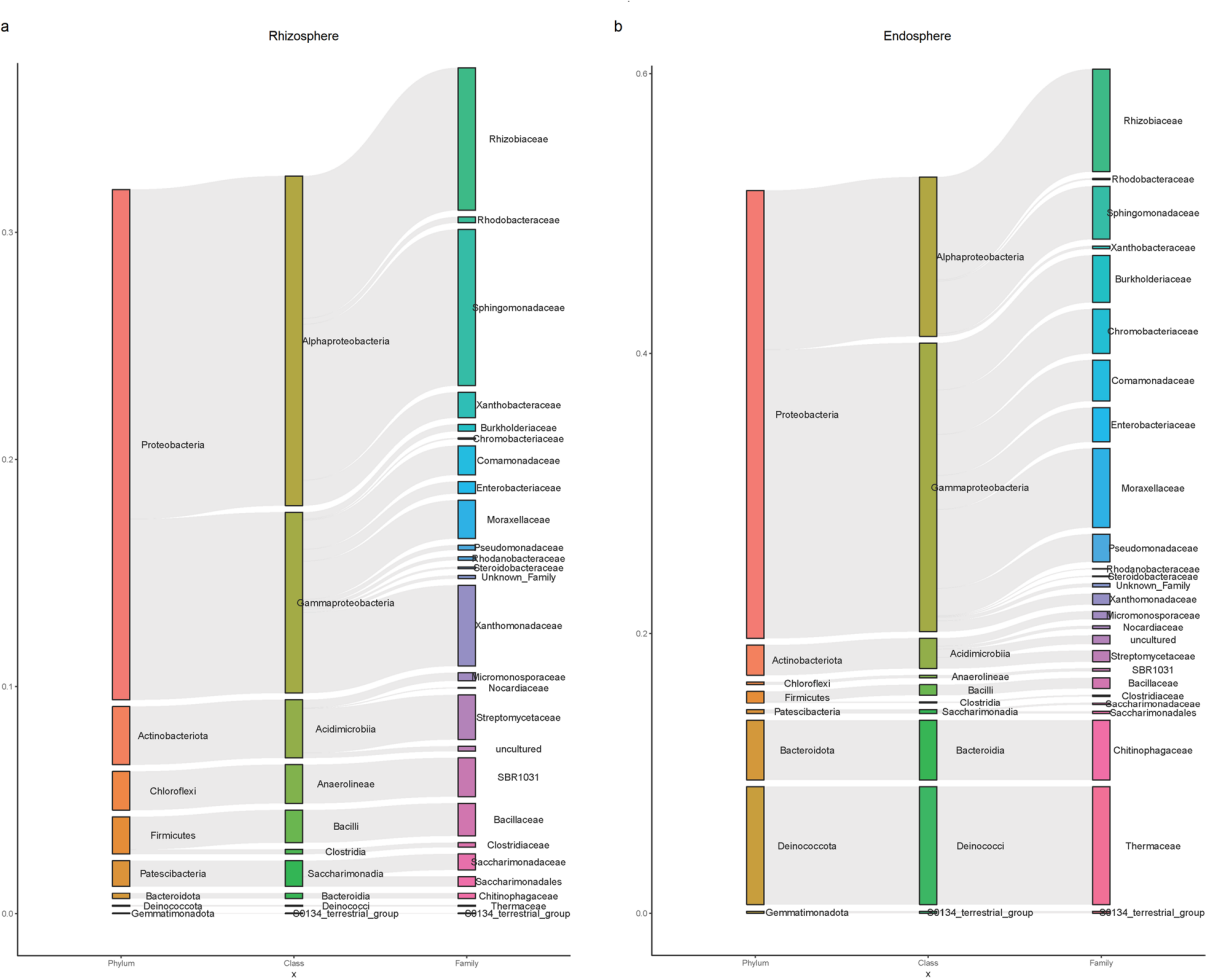
Core microbiome associated with the rhizosphere (**a**) and roo endosphere (**b**) of grafted apple. The heights of the rectangles indicate the average relative abundance of taxa

The number of common ASVs in the rhizosphere and root endosphere of four different rootstocks was 1379 and 202, respectively (Fig. 2a-b). These common ASVs revealed a Core ASVs group (*n* = 63) in the root systems of grafted apple (Fig. 2c). The core ASVs group counted for 29.8% (the rhizosphere) and 48.3% (the root endosphere) of the total sequences (Supplementary Table S2). The core microbiome consisted of Proteobacteria (*n* = 41), Actinobacteria (*n* = 10), Bacteroidota (*n* = 3), Patescibacteria (*n* = 3), Chloroflexi (*n* = 2), Firmicutes (n = 2), Deinococcotan (n = 1) and Gemmatimonadota (*n* = 1), accounting for 22.47–31.98%, 2.15–2.56%, 0.23–4.25%, 0.28–1.13%, 0.20–1.70%, 0.83–1.63%, 0.05–8.43% and 0.02–0.14% of the total sequences (Fig. 3). The core microbiome contains the following bacterial families: Micromonosporaceae, Nocardiaceae, Streptomycetaceae, Chitinophagaceae, SBR1031, Thermaceae, Bacillaceae, Clostridiaceae, S0134_terrestrial_group, Saccharimonadaceae, Saccharimonadales, Rhizobiaceae, Rhodobacteraceae, Sphingomonadaceae, Xanthobacteraceae, Burkholderiaceae, Chromobacteriaceae, Comamonadaceae, Enterobacteriaceae, Moraxellaceae, Pseudomonadaceae, Rhodanobacteraceae, Steroidobacteraceae, Xanthomonadaceae and Some unclassified families (Fig. 3). We found that the average relative abundance of Xanthomonadaceae, Sphingomonadaceae, and SBR1031 in the rhizosphere was significantly higher than that in the root endosphere (*P* < 0.05), while Burkholderiaceae, Rhizobiaceae, Moraxellaceae, Chromobacteriaceae, Chitinophagaceae Comamonadaceae, Enterobacteriaceae, Pseudomonadaceae and Thermaceae of the root endosphere was significantly higher than in the rhizosphere (*P* < 0.05) (Fig. 3).

### Composition and difference of bacterial communities associated with the root systems of grafted apple

**Fig. 4:**
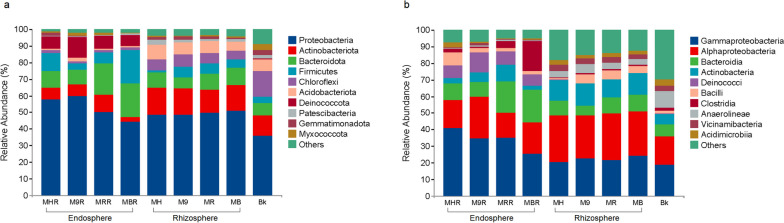
Relative abundance of the rhizosphere and root endosphere bacteria from grafted apple at the phylum (**a**) and class (**b**) levels. Only phyla and classes with relative abundance higher than 1% were shown in at least one sample, and relative abundance less than 1% were classified as “others”. MH, M9, MB and MR represent the rhizosphere from *Malus hupehensis* Rehd., M9T337, *Malus baccata* Borkh. and *Malus robusta* Rehd., respectively. MHR, M9R, MBR and MRR represent the root endosphere from *Malus hupehensis* Rehd., M9T337, *Malus baccata* Borkh. and *Malus robusta* Rehd., respectively. Bk represents bulk soil

According to the taxonomy annotation, the ASVs were classified into 45 phyla, 149 classes, 383 orders, 687 families, and 1619 genera. Proteobacteria (36.02–59.68% of the total sequence) were dominated phyla in the rhizosphere and root endosphere of apple grafted on four different rootstocks, followed by Actinobacteria (7.0–16.5%), Bacteroidetes (6.97–16.50%), and Firmicutes (1.43–20.24%) (Fig. 4a). The relative abundance of Chloroflexi, Acidobacteriota, Deinococcota, Patescibacteria, Gemmatimonadota, and Myxococcota exhibited more than 1% in at least one sample. At the class level, the dominant classes were Gammaproteobacteria (18.94–41.12%), Alphaproteobacteria (16.72–28.20%), Bacteroidia (6.05–19.91%), and Actinobacteria (3.33–12.94%) (Fig. 4b). Furthermore, compared with the root endosphere, the relative abundance of the dominant bacteria in the rhizosphere of four different rootstocks were similar at both phylum and class levels (Fig. 4).

**Fig. 5:**
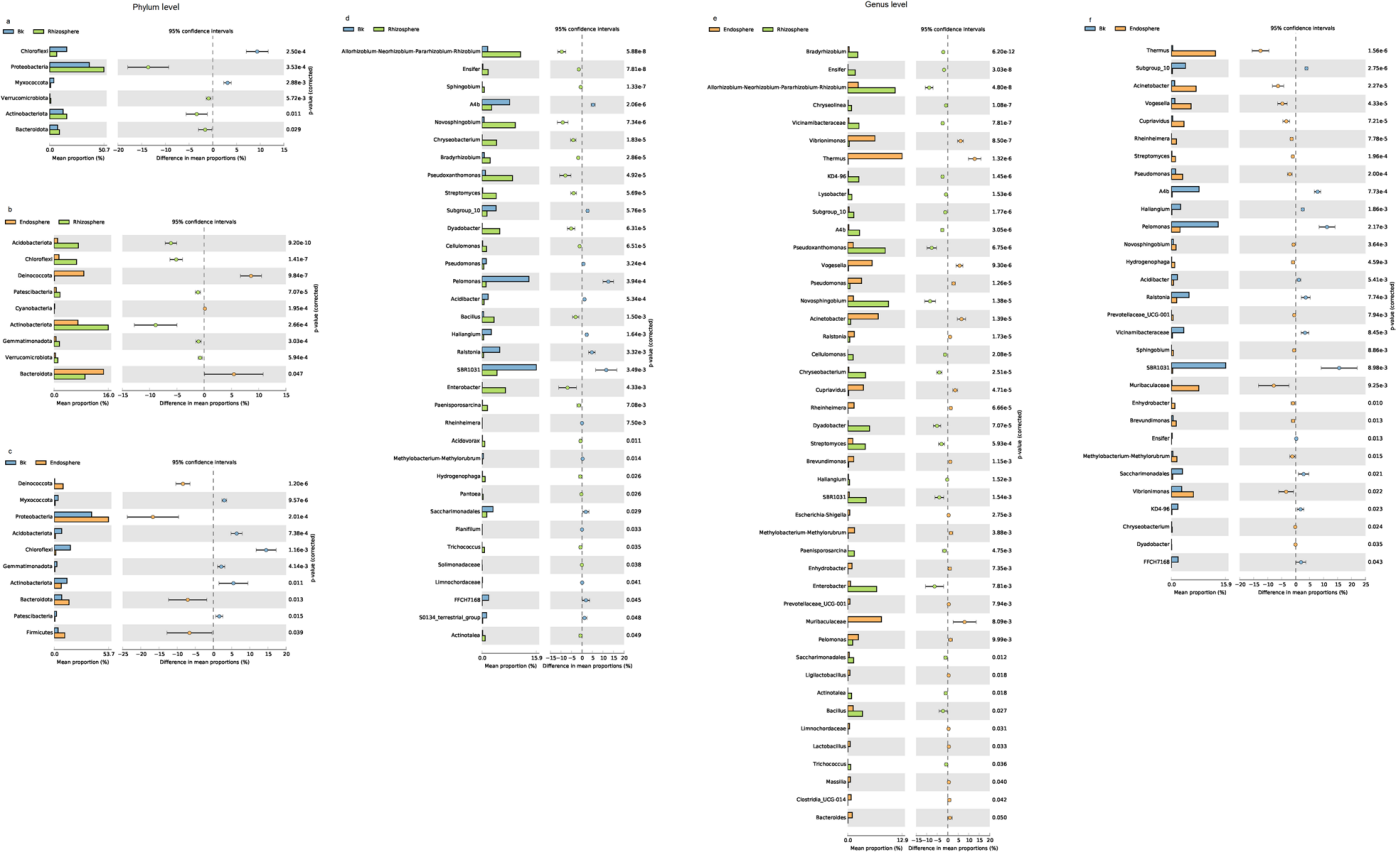
Differential abundance between bulk soil, rhizosphere, and root endosphere bacteria at the phylum (**a**, **b**, and **c**) and genus (**d**, **e**, and **f**) levels. Only phyla and classes with relative abundance higher than 1% were shown in at least one sample

Bacterial taxa distributions demonstrated significant differences in the three microhabitats (bulk soil, rhizosphere, and root endosphere). Compared with the bulk soil and root endosphere, Actinobacteriota, Verrucomicrobiota, *Allorhizobium-Neorhizobium-Pararhizobium-Rhizobium*, *Novosphingobium*, *Pseudoxanthomonas*, *Enterobacter*, *Streptomyces*, *Dyadobacter*, *Bacillus*, and *Chryseobacterium* had the highest abundance in the rhizosphere (*P* < 0.05) (Fig. 5). The abundance of Bacteroidota, Firmicutes, Proteobacteria, *Sphingomonas*, *Klebsiella*, *Acidovorax*, *Hydrogenophaga*, *Aestuariicella*, *Sphingobium*, *Solimonadaceae*, *Pantoea*, and *Kosakonia* were progressively increased in the root-soil interface (from the bulk soil to the rhizosphere to the root endosphere), finally enriched significantly in the root endosphere (*P* < 0.05) (Fig. 5). Similarly, the abundance of Acidobacteriota, Chloroflexi, Gemmatimonadota, *SBR1031*, *A4b*, *Vicinamibacteraceae*, *Subgroup 10*, *Saccharimonadales*, *Lysobacter*, *Chryseolinea*, *Haliangium*, and *FFCH7168* were progressively decreased, finally depleted significantly in the root endosphere (*P* < 0.05) (Fig. 5). In conclusion, the bacterial community of the bulk soil was not only filtered, assembled and enriched by the rhizosphere, but also further selected and recombined by the root endosphere.

### Diversity of bacterial communities associated with the root systems of grafted apple

**Fig. 6:**
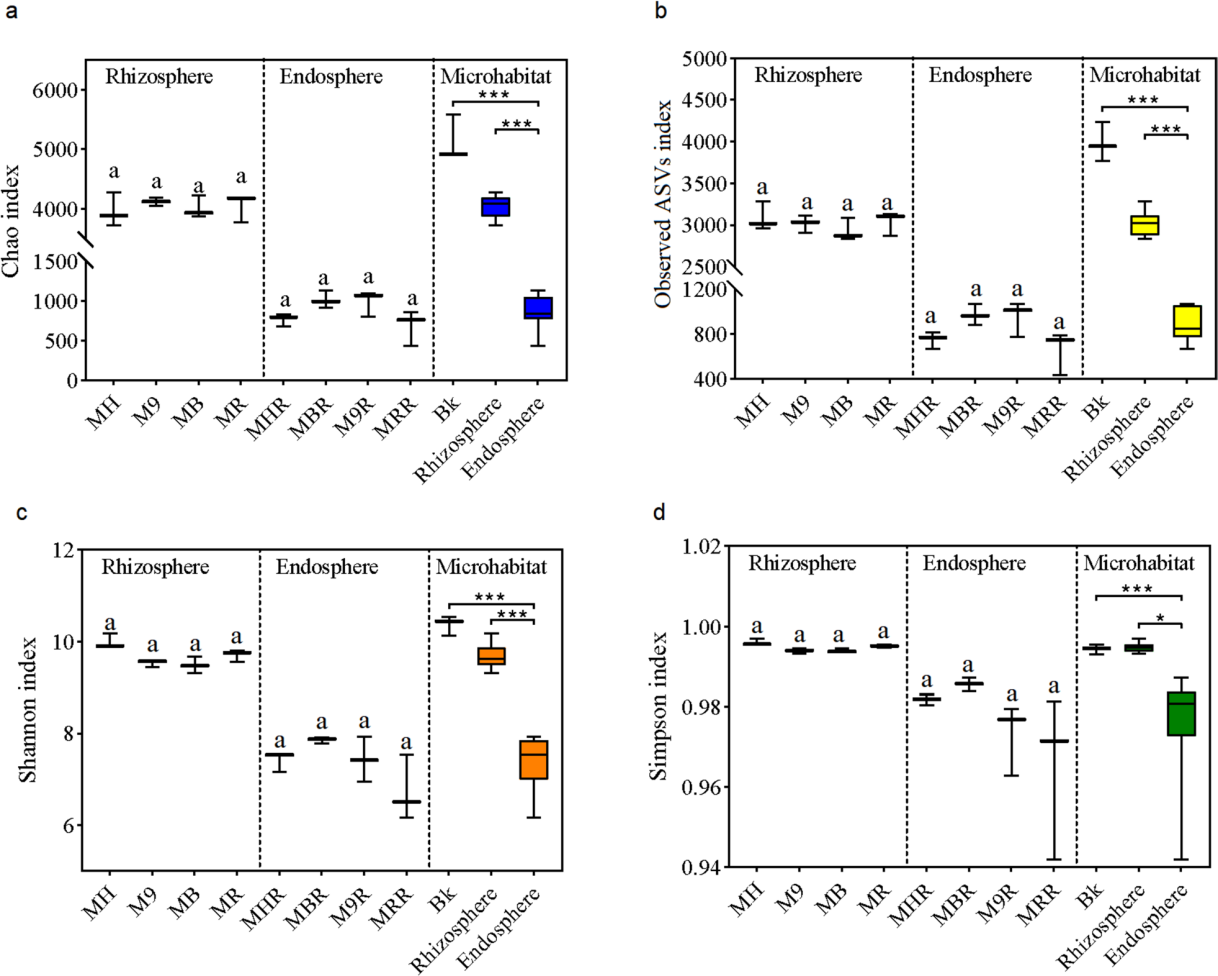
Richness (**a**, **b**) and diversity index (**c**, **d**) of the bacterial communities associated with the rhizosphere and root endosphere of grafted apple. Different lowercase letters indicate significant differences by Kruskal-Wallis test (*P* < 0.05). Stars indicate significant differences by the Mann-Whitney-Wilcoxon test. (* *P* < 0.05, ** *P* < 0.01, *** *P* < 0.001). MH, M9, MB and MR represent the rhizosphere from *Malus hupehensis* Rehd., M9T337, *Malus baccata* Borkh. and *Malus robusta* Rehd., respectively. MHR, M9R, MBR and MRR represent the root endosphere from *Malus hupehensis* Rehd., M9T337, *Malus baccata* Borkh. and *Malus robusta* Rehd., respectively

The α-diversity of the bacterial community expressed as richness (observed ASVs and Chao1 index) and diversity (Shannon index and Simpson index) in the rhizosphere and root endosphere of grafted apple (Fig. 6). The Chao, observed ASVs, Shannon and Simpson index did not observe a significant difference in the rhizosphere and root endosphere of four different rootstocks (Fig. 6). Besides, the richness and diversity of the rhizosphere bacteria were significant higher than that of the root endosphere regardless of the rootstock types (Fig. 6). In the three root microhabitats, the α-diversity index showed a trend of the bulk soil > rhizosphere > root endosphere.

**Fig. 7:**
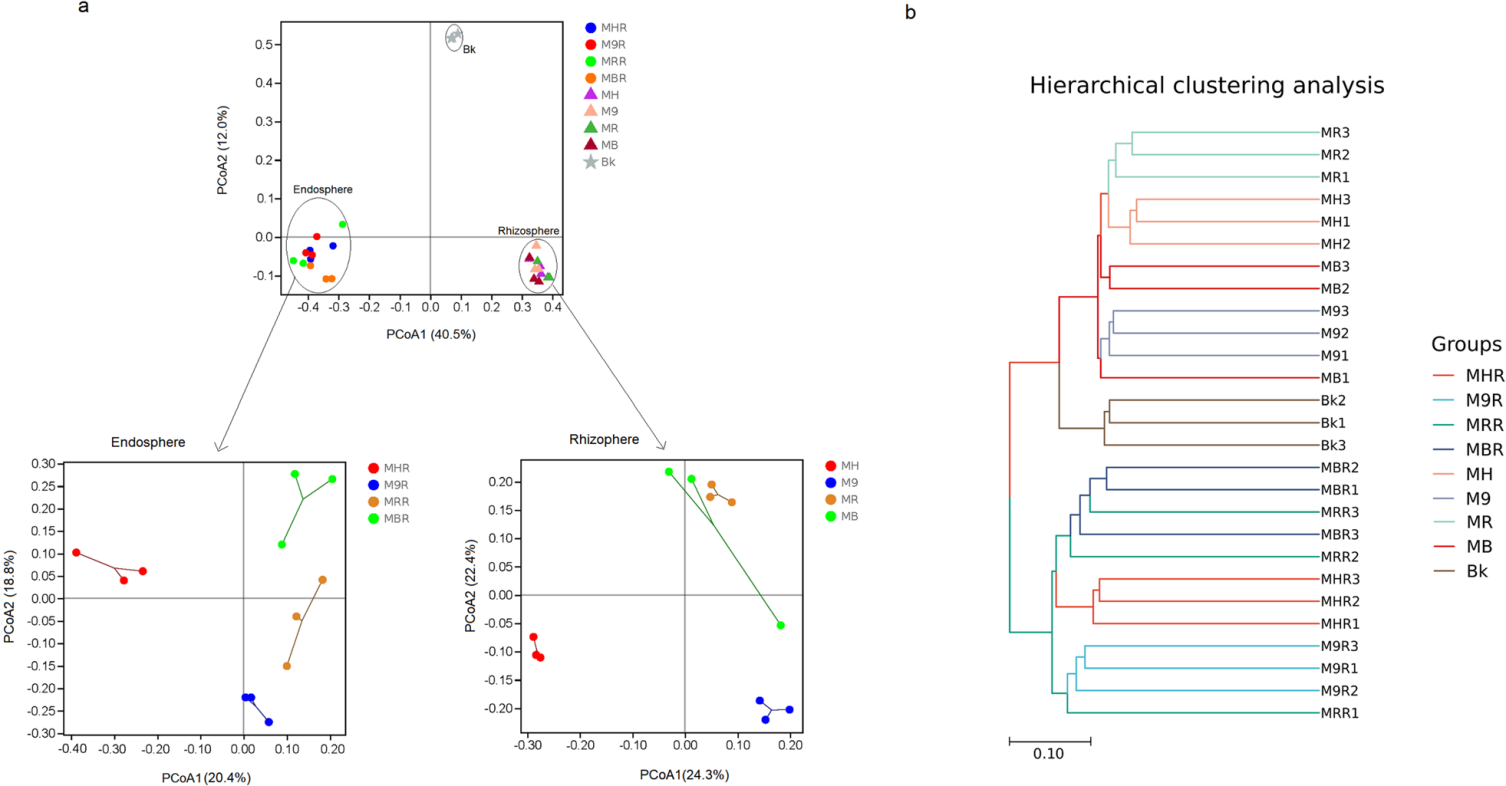
Principal coordinate analysis (PCoA) (**a**) and Clustering analysis (**b**) of the bacterial communities associated with the rhizosphere and root endosphere of grafted apple. MH, M9, MB and MR represent the rhizosphere from *Malus hupehensis* Rehd., M9T337, *Malus baccata* Borkh. and *Malus robusta* Rehd., respectively. MHR, M9R, MBR and MRR represent the root endosphere from *Malus hupehensis* Rehd., M9T337, *Malus baccata* Borkh. and *Malus robusta* Rehd., respectively. Bk represents bulk soil

We used PCoA analysis and clustering analysis based on Bray-Curtis distance measures to estimate β-diversity (Fig. 7). The PCoA demonstrated that all the samples were separated by the PCoA1 axis (40.5%) and clustered into three groups of the bulk soil, rhizosphere, and root endosphere, and different rootstock types were also distinctly separated (Fig. 7a). But the PerMANOVA test based on the Bray-Curtis distance measures showed that the bacterial community structure was not significantly (*P* > 0.05) different among rhizosphere and root endosphere of four different rootstocks (Supplementary Table S3). Similar results were also supported by the cluster tree (Fig. 7b). The result showed that the rhizosphere of four different rootstocks and bulk soil were divided into a cluster, and then clustered into two different clusters. However, the root endosphere of four different rootstocks was separated into a single cluster. These results indicated that variation of the bacterial community was mainly driven by the different root microhabitats (bulk soil, rhizosphere, and root endosphere), followed by rootstock types.

### Co-occurrence networks of bacterial communities associated with the root systems of grafted apple

**Fig. 8:**
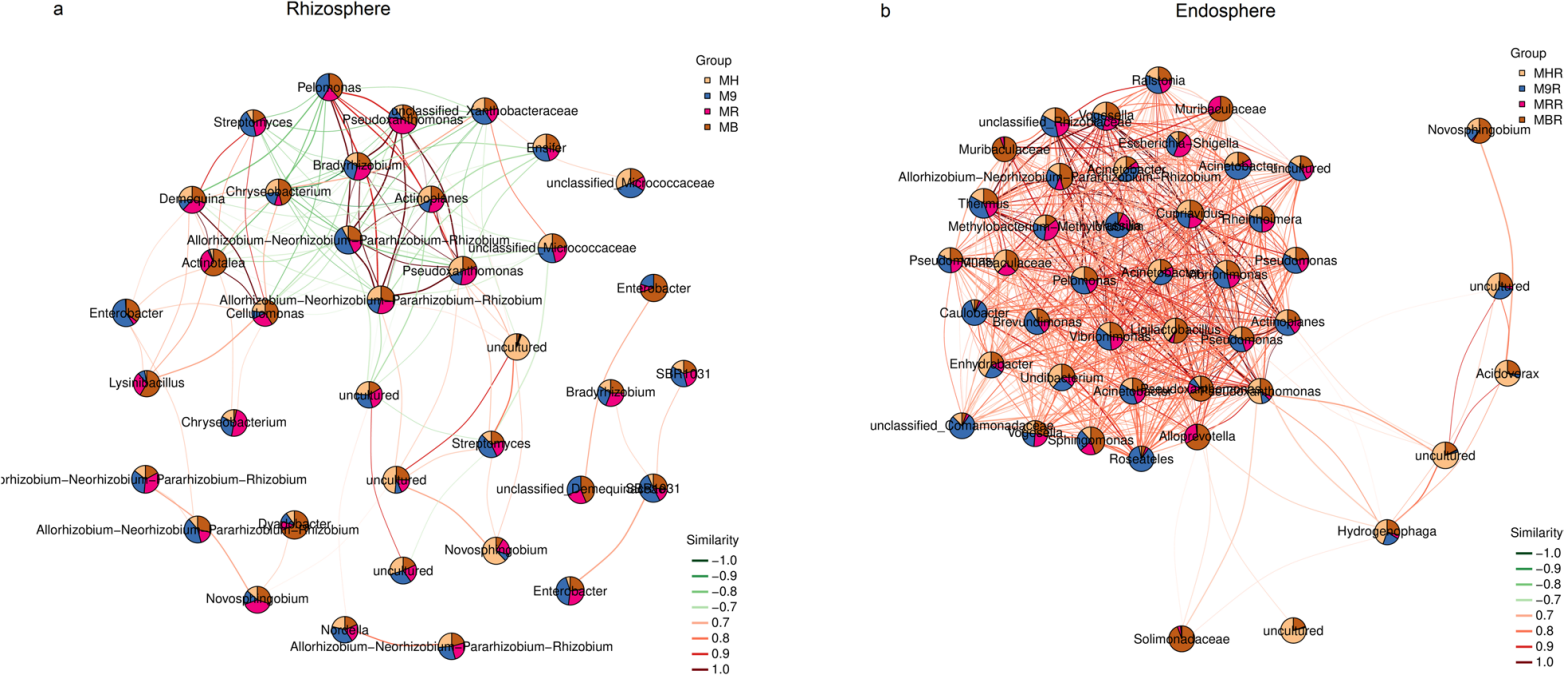
Co-occurrence networks of the bacterial communities in the rhizosphere (**a**) and root endosphere (**b**) of grafted apple. The nodes are colored according to group and node area of the edges are correlated to the abundance of the taxa. Different color edges represent co-exclusion and co-occurrence, respectively

In co-occurrence networks analysis, we explored the differences of bacterial networks and identify the keystone nodes (species) in the rhizosphere and root endosphere of grafted apple (Fig. 8). This was done to reduce the level of complexity of the presented data. Notably, the bacterial interactions in root endosphere were dominated by co-occurrence i.e. positive interactions, while the bacterial interactions in rhizosphere exhibited a mixture of co-occurrence and co-exclusion i.e. positive and negative interactions (Fig. 8). The rhizosphere network networks were more complex and contained a higher number of nodes than root endosphere networks (Table 1). Although the size of the the rhizosphere network (avg. number of nodes = 3089, avg. no, edges = 164,450) was larger than the root endosphere network (avg. no. nodes = 1307, avg. no. edges = 35,005), the root endosphere network density was much greater for bacteria (Average = 0.041) than rhizosphere (Average = 0.034). This indicates that the members of the bacterial community in root endosphere had a much higher tendency to interact with each other than that in root endosphere (Fig. 8, Table 1). Compared with the root endosphere network, the rhizosphere network seems to be better connected, with a degree equal to the grassland network, a relatively high closeness centrality, high betweenness centrality, and low average path length (Fig. 8, Table 1). Using Zi and Pi value, we found that 44 and 21 ASVs were classified as keystone nodes (species) in the rhizosphere and root endosphere, respectively (Supplementary Table S4). In the rhizosphere network, the keystone taxa were mainly from Proteobacteria, Actinobacteriota, Bacteroidota, and Acidobacteriota. Moreover, 20 network hubs (Zi > 2.5 and Pi > 0.62) with 9.99% of the total sequences was found, most belonging to the *Dyadobacter* and *Novosphingobium*. Alphaproteobacteria, Gammaproteobacteria, Acidimicrobiia, and Chloroflexia were mainly keystone taxa in the root endosphere network, thereinto, *Sphingomonas* and Rhizobiaceae were the two most abundant keystone nodes. Interestingly, the keystone taxa had mostly low abundances (relative abundance less than 1%) in the rhizosphere and root endosphere network, accounting for 6.84 and 2.79% of the total sequences, respectively (Supplementary Table S4).

**Table 1:** Topology parameters associated with the constructed co-occurrence networks for the rhizosphere and root endosphere of grafted apple

	Rhizosphere	Endosphere
Average nearest neighbor degree	107.44	54.49
Average path length	1.993	2.065
Betweenness centrality	3,793,224.72	977,348.59
Closeness centrality	24.238	29.388
Degree centralization	112,827	31,674.6
Density	0.034	0.041
Diameter	3	3
Transitivity	0.034	0.041
No. nodes	3089	1307
No. edges	164,450	35,005
Modularity	0.084	0.117

## Discussion

To describe the bacterial community composition of the bulk soil, rhizosphere and root endosphere from apple grafted on the four different rootstocks, we used next-generation sequencing of the 16S rRNA gene to avoid the influence of culture conditions and more completely reveal the characteristics of the bacterial community in a specific environment.

Microorganisms could multiply in specific niches and form community structures. Which was contributed by the interaction between the microbiomes and the niches. Such a result mainly depended on the host genotype and the environmental conditions of the niche [21, 31]. Previous studies had indicated that the soil harbours abundant microbial resources, the majority of the root endosphere and rhizosphere microbiomes originated in the soil [32]. The bulk soil microorganisms were attracted by host genotype-dependent root induction factors (such as root exudates), through further adjustment and assembly of the root tissue, thereby established a unique root-associated microbiota, i.e. the rhizosphere and root endosphere [33]. According to our findings, bacterial richness and diversity showed the bulk soil > rhizosphere > root endosphere (Fig. 6). The number of unique and common ASVs in the rhizosphere was significantly higher than the root endosphere (Fig. 2a-b). These results showed that the rhizosphere and root endosphere microbiomes were regulated and filtered by the root systems of grafted apple. On the other hand, as to the richness of the rhizosphere in four different rootstocks, there was no significant difference (Fig. 6a-b), as to the diversity of the root endosphere, we achieved the same conclusion (Fig. 6c-d). Besides, the PCoA showed that the bacterial communities of the bulk soil, rhizosphere and root endosphere were divided into three independent groups, which were not affected by rootstock types (Fig. 7a). These results were also supported by cluster analysis (Fig. 7b). These analyses revealed significant differences of the bacterial communities in the three microhabitats, while rootstock types had a slight effect on the rhizosphere and root endosphere bacteria of grafted apple.

The taxonomic composition analysis showed that the most abundant bacterial phyla were Proteobacteria, Acidobacteria, and Bacteroidetes in the rhizosphere and root endosphere of grafted apple (Fig. 4a). The members of Proteobacteria had the characteristics of rapid growth and strong adaptability, and it iwas the most common dominant phyla of many plant root-associated bacteria [34–36]. Acidobacteria also was the major bacterial population in the soil, and it could produce antibacterial or antifungal activity compounds to enhance plant health [37]. The members of Bacteroidetes belonged to copiotrophic bacteria, the rich organic resources in the rhizosphere and root endosphere allowed them to multiply abundantly [38]. In the three different microhabitats (bulk soil, rhizosphere, and root endosphere), we found that Chloroflexi, Acidobacteria, Anaerolineae and Gemmatimonadota were gradually depleted in the root-soil interface, while Bacteroidota, Firmicutes, and Proteobacteria were gradually enriched (Fig. 5). Interestingly, The core microbiome also showed the same trend of enrichment and depletion in the rhizosphere and root endosphere (Fig. 3). These changes distinguished root-associated microbiomes from the bulk soil, forming three slowly differentiated microbiomes niche through the filtration and assembly of the root system.

Our data indicated that only 63 ASVs out of 24485consistently present in the rhizosphere and root endosphere of grafted apple (Fig. 2c), but the abundance counted for 29.8% (the rhizosphere) and 48.3% (the root endosphere) of the total sequences (Supplementary Table S2). These core ASVs, classified up to family level, contained Micromonosporaceae, Nocardiaceae, Streptomycetaceae, Chitinophagaceae, SBR1031, Thermaceae, Bacillaceae, Clostridiaceae, S0134_terrestrial_group, Saccharimonadaceae, Saccharimonadales, Rhizobiaceae, Rhodobacteraceae, Sphingomonadaceae, Xanthobacteraceae, Burkholderiaceae, Chromobacteriaceae, Comamonadaceae, Enterobacteriaceae, Moraxellaceae, Pseudomonadaceae, Rhodanobacteraceae, Steroidobacteraceae, Xanthomonadaceae (Fig. 3). Most of this core microbiome provided beneficial services for the growth of host plants. Many species of the Burkholderiaceae, Xanthomonasceae, Moraxellaceae and Rhizobiaceae were plant-growth-promoting bacteria, which could produce plant hormones (such as indole-3-acetic acid, Cytokinin and 1-aminocyclopropane-1-carboxylic acid deaminase, inhibit the spread of pathogens, induce systemic resistance in plants and promote N_2_-fixation, phosphate solubilization to promote plant growth [9, 12, 39, 40]. Several species of Chitinophagaceae had been shown to be able to secrete active enzymes that degrade carbohydrates [41]. The member of Sphingomonadaceae could promote nitrogen fixation and the degradation of aromatic compounds [42, 43]. The rhizosphere and root endosphere regulated by the host root could identify and select beneficial microorganisms for their growth. The core microbiome was the result of interactions and adaptation between the rhizosphere, root endosphere and microorganisms. This result should be beneficial to the healthy growth of the apple root system and the establishment of an underground microbial network.

Microorganisms were not isolated in the microbial community, but through the interconnection to establish a complex association network under specific assembly conditions, then maintained the host-microbial homeostasis [44, 45]. Here, we explored the co-occurrence patterns in the rhizosphere and root endosphere of grafted apple. Compared with the root endosphere, we found that the rhizosphere network had a higher scale and complexity based on the topological properties (Fig. 8, Table 1). Only bacteria that were able to pass through root cortical and endodermis can continue to colonize the endothelial layer [5, 46]. Therefore, the root endosphere environment had a stronger filtering effect on microorganisms than the rhizosphere. This filtering effect and the root endosphere microbiomes were separated by internal root tissue (such as xylem, phloem, pericycle, and vascular tissues), which not only reduced the diversity of the root endosphere microbiomes, but also weakened the interaction and connection between each other. These factors contributed to the less complexity of bacterial networks in the root endosphere.

The relationship network established by microorganisms through cooperation, competition and symbiosis plays a crucial role in the microbial community composition [47, 48]. Alphaproteobacteria, Gammaproteobacteria, Bacteroidetes and Actinobacteria occupied most of the nodes and edges in the rhizosphere and root endosphere bacterial networks (Supplementary Table S4, Fig. 8). Interestingly, these bacteria were also the dominant phyla or classes in the root systems of grafted apple (Fig. 4). These results suggested that the bacterial community and the root systems adapt and select each other, and the selected bacteria will actively participate in the interconnection of the network. Keystone nodes were highly connected taxa in the microbial community network, and the disappearance or decrease of these might reduce the connectivity and complexity of the network [49]. A similar trend, the keystone species in the network were directly affected by changes in the external environment condition, and through the interaction between microorganisms, the effects of environmental changes were transmitted to the entire microbial network [50]. We found that the keystone species belonged to Proteobacteria, Actinobacteriota and Bacteroidetes in the rhizosphere and root endosphere bacterial networks (Supplementary Table S4). *Dyadobacter* and *Novosphingobium* were the two most abundant network hubs (Zi > 2.5 and Pi > 0.62) in the rhizosphere network, and *Sphingomonas* and Rhizobiaceae were the two most abundant keystone nodes in root endosphere bacterial networks (Supplementary Table S4), which might play an important role in maintaining the stability and structure of the bacterial community in rhizosphere and root endosphere of grafted apple. *Dyadobacter*, *Novosphingobium*, *Sphingomonas* and Rhizobiaceae were plant growth-promoting bacteria that could resist various pathogens and produce phytohormones [51–53]. *Dyadobacter* also had potential to promote plant growth by fixing atmospheric N_2_ and making it available to plant [54]. *Novosphingobium* had been reported to promote the growth of tobacco by increasing nutrient uptake, and improving root morphology [55]. *Novosphingobium* and *Sphingomonas* were also known to induce root growth via the production of gibberellinsand Indole-3-acetic acid [56, 57]. As is known to all, Rhizobiaceae could be able to colonize the roots and they can fix N_2_ from the atmosphere, providing leguminous plants with ammonia (NH_3_) as an essential nutrient. Root colonization by these microorganisms might result in nitrogen fixation, enhanced nutrient acquisition from the soil, and improved nitrogen use efficiency [58]. In addition, most of the keystone species were low abundance taxa (Supplementary Table S4). A recent study showed soil ecosystem functions were driven by rare rather than abundant microbial taxa under long-term greenhouse cultivation [59]. Taken together, high abundance did not mean high connectivity, some low-abundance species with high connectivity might play a significant role in maintaining the microbial network structure and ecosystem stability [30, 52].

## Conclusions

In this study, the hierarchical filtration pattern of the bacterial community was demonstrated by the enrichment and depletion of bacterial phylum and class levels in the root-soil interface (from the bulk soil to the rhizosphere to the root endosphere), as well as a progressive decrease of bacterial α-diversity. Interestingly, the same pattern was found in the core microbiome of grafted apple. Furthermore, the core microbiome containing only 63 ASVs an unmatched abundance proportion, accounting for 29.8% (the rhizosphere) and 48.3% (the root endosphere) of the total sequence. In contrast to keystone nodes of the network, they mostly were low-abundance species, accounting for 6.84% (the rhizosphere) and 2.79% (the root endosphere) of the total sequences. This suggests that low-abundance species may play an important role in connecting high-abundance species.

### Supplementary Information

**Additional file 1: Table S1.** Raw data for the rarefaction curves of the number of ASVs for each sample. **Table S2.** Average relative abundance of core ASVs in the rhizosphere and root endosphere. **Table S3.** The PerMANOVA test based on the Bray-Curtis distance measures in the rhizosphere and root endosphere of grafted apple. **Table S4.** Keystone nodes of the bacterial networks in the rhizosphere and root endosphere of grafted apple.

## Data Availability

All data generated or analyzed during this study are included in this article and its supplementary information fles. The bacterial raw sequences used in data analysis have been submitted in the NCBI Sequence Read Archive under accession number SRP280070 (https://www.ncbi.nlm.nih.gov/sra/?term=SRP280070).
